# MicroRNA-503 and the Extended MicroRNA-16 Family in Angiogenesis

**DOI:** 10.1016/j.tcm.2012.05.003

**Published:** 2011-08

**Authors:** Andrea Caporali, Costanza Emanueli

**Affiliations:** Laboratory of Vascular Pathology and Regeneration, School of Clinical Sciences, University of Bristol, Bristol BS2 8HW, UK

## Abstract

MicroRNAs (miRs) are post-transcriptional inhibitory regulators of gene expression acting by direct binding to complementary messenger RNA (mRNA) transcripts. Recent studies have demonstrated that miRs are crucial determinants of endothelial cell behavior and angiogenesis. We have provided evidence of the prominent role of miR-503 in impairment of postischemic reparative angiogenesis in the setting of diabetes. Because miR-503 belongs to the miR-16 extended family of miRs, in this review, we describe the cardiovascular functions of miR-503 and other members of the miR-16 family and their impact on angiogenesis.

## Introduction

MicroRNAs (miRs) are small, noncoding RNAs that regulate gene expression, mainly by binding 3' untranslated regions (3' UTRs) of messenger RNA (mRNA) transcripts. Regulation of miRs is an emerging feature in developmental biology, cancer, and cardiovascular disease (Sayed and Abdellatif 2011). miRs are predicted to target more than 50% of all human protein-coding genes, thus inducing translational repression, mRNA degradation, or mRNA instability. Hence, accurately determining miR targets is essential for understanding the functional roles of miR in physiology and diseases.

Blood vessel formation by angiogenesis is a complex multistep process that requires control and coordination of endothelial cell (EC) behavior as well as the contribution of pericytes and other intra- and extravascular supportive cells. Commonly recognized as “cell embedded within the vascular basement membrane” and identified by several molecular markers (alpha smooth muscle actin, nonmuscle myosin, tropomyosin, desmin, nestin, platelet-derived growth factor receptor-β [PDGFR-β], aminopeptidases A and N [CD13], sulfatide, and nerve/glial antigen-2 proteoglycan [Díaz-Flores et al. 2009]), pericytes play an essential role in the stabilization and maturation of new vascular networks. PDGF-B, released from ECs undergoing angiogenic remodeling, acts as a chemoattractant for comigrating pericytes that express PDGFR-β. Recruited pericytes are incorporated into the wall of immature vessels and establish direct cell-cell contact with ECs. Interactions occurring between pericytes and ECs are strengthened by several molecules, such as transforming growth factor-β (TGF-β), vascular endothelial growth factor (VEGF), and angiopoietins, and by signaling pathways involving Notch and ephrins (Armulik et al. 2011).

In stable vessels, ECs typically form a monolayer of quiescent cells that lines the luminal surface of vascular tubes. In response to proangiogenic stimuli, ECs loosen their cell-cell contacts, activate proteases that degrade the surrounding basement membrane, and acquire extensively invasive and motile behavior to initiate new blood vessel sprouting, followed by further vascular morphogenesis and maturation (Potente et al. 2011). Emerging evidence suggests that miRs might contribute to the fine-tuning of the genes involved in the angiogenic process.

Disrupted balance of angiogenesis contributes to the pathogenesis of numerous disease states (Carmeliet and Jain 2011). For example, uncontrolled angiogenesis favors tumor growth and metastasis. Although it is desirable to block the growth of new blood vessels in these circumstances, the controlled stimulation of angiogenesis is beneficial in ischemic conditions, characterized by impaired local blood supply. Considering the potential clinical benefits of therapeutically manipulating blood vessel growth and blood flow, the mechanisms regulating the angiogenetic process have formed a major focus for vascular research during the past two decade.

## miR-503 Belongs to the miR-16 Family

The 5'-end portion (5' UTR) of miRs, also known as the “seed region,” is a particularly important determinant of miR function (Lewis et al. 2005). miRs with the sequence AGCAGC (AGCx2), starting at the second nucleotide from the 5' end of their mature form, belong to the “canonical” miR-16 family. Members of this family are miR-15a/b, miR-16, miR-195, miR-424, and miR-497. Moreover, based on the presence of AGCx2 motif starting at the first nucleotide in seed sequence, miR-103, miR-107, and miR-646 could be also included in an “extended” miR-16 family (Finnerty et al. 2010). The seed region of miR-503 differs only at nucleotide 8 from the seed region of the canonical miR-16 family: This nucleotide is a guanosine in miR-503 and an adenosine in the miR-16 family. This leads to an overlap between target genes because 8-mer sites (positions 2-8 of the mature miR followed by an “A”) for miR-503 are recognized as 7-mer-A1 (positions 2-7 of the mature miR followed by an “A”) sites by canonical miR-16 family members and vice versa (Rissland et al. 2011) (Table 1). In agreement with the knowledge that the AGC2x motif near the miRs' 5' end controls the miR target specificity, there is evidence that different members of the miR-16 family, including miR-503, regulate overlapping miR targets (Forrest et al. 2010, Joglekar et al. 2007).

miR-503 is an intragenic miRNA clustered with miR-424 on chromosomal location Xq26.3 (Griffiths-Jones et al. 2008). miR-424 has been implicated in regulation of angiogenesis (Ghosh et al. 2010, Chamorro-Jorganes et al. 2011). miR-503 and miR-424 are separated by 383 bases on the genome and are likely derived from the same primary transcript as a pair of hairpins and to have related seed sequences. A further five miRs (miR-542-5p, miR-542-3p, miR-450a, miR-450b-5p, and miR-450b-3p) are within 7 kb of the miR-424–miR-503 cluster and can be transcribed from the same primary transcript. It is not known if these additional five miRs are expressed in vascular cells or involved in angiogenesis. Members of the miR-16 family are also intragenic miRNA and expressed from different loci as pairs of hairpins, closely spaced with linker regions of 150-200 nucleotides.

Considering the seed sequence similarities and the genomic organization, it is possible to classify miR-503 as part of the extended miR-16 family (Table 2).

## miR-503 Expression and Functions

miR transcription is controlled through the same regulatory machinery as for the protein coding genes (Lee et al. 2004). Understanding the mechanisms regulating the activation or repression of miR transcription requires locating their core promoter regions using promoter prediction methods (Zhou et al. 2007). Moreover, Marson et al. (2008) found that the transcription starting sites (TSSs) within the promoter regions of miRs are determined by the presence of trimethylated histone H3 at its lysine 4 residue (H3K4me3). Using bioinformatic approaches, including the extraction of promoter regions for miR genes, computational prediction of transcription factor binding sites (TFBSs) to establish TFs/miR associations, and the use of time course expression data for TFs and miRs, Schmeier et al. (2009) identified the following TFs that bind in the promoter region of miR-424: RUNX1, E2F3, SP3, YY1, NFE2L2, CREB1, ATF2, USF2, ELK1, CEBPB, and HOXA4. Because pre-miR-503 is transcribed together with pre-miR-424 as one transcript, it is likely that miR-503 expression is subjected to the same transcriptional regulation of miR-424. The mechanisms of miR-503 transcription and maturation have not yet been demonstrated, and more “wet” experiments are needed to verify this hypothesis.

miR-503 was first identified in human retinoblastoma tissues using the miR microarray technique (Zhao et al. 2009). Moreover, miR-503 expression was found upregulated in human parathyroid carcinomas (Corbetta et al. 2010) and in adenocortical carcinomas (Tömböl et al. 2009)). In monocytes differentiating toward macrophages, miR-424 and miR-503 are produced as a polycistronic message and induce G1 arrest by targeting an overlapping set of cell cycle regulators (Forrest et al. 2010). In line with this, induction of G1 phase cell cycle arrest is critical for the differentiation of myoblasts into myotubes. Moreover, miR-424 and miR-503 are induced during myogenesis and promote cell cycle arrest through cdc25A degradation (Sarkar et al. 2010). Hence, these two miRs appear to be part of the differentiation processes. In addition, miR-503 expression increases in response to serum starvation in mesenchymal stem cells (Nie et al. 2011), NIH-3T3 cells (Rissland et al. 2011), and ECs cultured in high glucose (Caporali et al. 2011). Interestingly, in NIH-3T3 G1 arrested by serum starvation, miR-503 returns to normal level upon cell cycle reentry (Rissland et al. 2011). miR-503 expression is also upregulated in response to growth arrest by cell contact inhibition. Rapid degradation during cell cycle reentry of miR-503 is dependent on its constitutive instability. Thus, miR-503 modulates the cell cycle and is itself dynamically regulated by the cell cycle.

Increased miR-503 in human umbilical vein ECs and human microvascular ECs results in cell cycle delay in G1, reduced cell proliferative and migratory capacity, and impaired EC networking capacities, suggesting an anti-angiogenic role for this miR (Caporali et al. 2011).

In vivo, miR-503 is upregulated in myocardial ECs from diabetic GK rats (Wang et al. 2009) and in ECs resident in ischemic limb muscles of diabetic mice (Caporali et al. 2011). It is also upregulated in ischemic limb skeletal muscles and plasma of diabetic patients with end-stage critical limb ischemia (Caporali et al. 2011).

## miR-503 Validated Target Genes

Recent studies have proposed that miR-503 might be a master regulator of the cell cycle (Rissland et al. 2011). Gene ontology analysis of genes affected by miR-503 identified those annotated as cell cycle regulators or involved in cell adhesion, migration, and angiogenesis processes (Caporali et al. 2011). Using prediction software, four genes were consistently identified as targets of miR-503: cell division cycle 25A (CDC25A), cyclin D1 (CCND1), cyclin D2 (CCND2), and cyclin E1 (CCNE1). By 3' UTR-luciferase assays, we validated CDC25A and CCNE1 as direct target genes of miR-503 (Caporali et al. 2011). CCND1 and CCND2 were validated as miR-503 targets by others (Jiang et al. 2009). The concept of miR-503 being involved in the cell cycle process has been further enforced by the finding of Forest et al. (2010) that mir-503 inhibits the expression of additional genes involved in cell division, the cell cycle, and mitosis, such as CCNE, CCNF, CDC14A, anillin, activating transcription factor 6 (ATF6), Argonaute 1 (Ago1), checkpoint kinase 1 (CHEK1), mitosis inhibitor protein kinase WEE1, and CDKN1A (p21). Seven of these targets are in common with miR-424 (Forrest et al. 2010). Interestingly, cell cycle genes involved in G1-S transition, such as CCDN1, CCNE1, and cdc25A, are targeted by most of the members of the extended miR-16 family (Liu et al. 2008, Rissland et al. 2011). Forrest et al. also showed that an increase in miR-503 downregulates miR-9-3, thus demonstrating reciprocal expressional regulation between miRs (Forrest et al. 2010). Because miRs can regulate other miRs, these interactions increase the complexity of gene regulation and are likely to be involved in regulatory processes that are currently unexplored.

## Extended miR-16 Family in Vascular Biology and Diabetes

Ghosh et al. (2010) reported that miR-424 is induced by hypoxia in ECs and promotes angiogenesis in vivo and in vitro through PU.1-dependent transactivation and direct inhibition of cullin 2 (CUL2), which increase HIF-1α levels. In contrast, Chamorro-Jorganes et al. (2011) demonstrated that overexpression of miR-424 or miR-16 reduces proliferation, migration, and angiogenic capacity of ECs in vitro and in a Matrigel plug implanted in mice. In agreement with the findings of Chamorro-Jorganes et al., miR-424 expressional inhibition using oligos anti-miR in human dermal microvascular ECs abnormally increases cell proliferation and promotes angiogenesis, possibly via increased levels of MEK1 or CCNE1 (Nakashima et al. 2010). Moreover, miR-424 and miR-16 reduce the expression of VEGF-A, VEGF receptor 2 (VEGFR2), and fibroblast growth factor receptor 1 (FGF-R1) by binding at 3' UTR sequences (Chamorro-Jorganes et al. 2011, Musumeci et al. 2011). Interestingly, either VEGF-A or basic FGF (bFGF) treatment upregulates expression of mature miR-16 and miR-424; thus, a regulatory loop between miR-16, miR-424, and the major proangiogenic growth factors exists. miR-16 is transcribed together with either miR-15a (miR-15a/-16-1 cluster) or miR-15b (miR-15b/-16-2 cluster). In line with data from Chamorro-Jorganes et al., we found that miR-16 and miR-15a are upregulated in proangiogenic circulating cells (PACs; previously known as endothelial progenitor cells) of patients with critical limb ischemia and that increased miR-15/16 impairs PAC functional capacities (Fortunato et al. 2012). Moreover, miR-15a, miR-15b, miR-16-1, miR-16-2, miR-424, and miR-497 were consistently found to be upregulated in cardiomyocytes during cardiac ischemia and heart failure (reviewed in Small et al. 2010). Hullinger et al. (2012) demonstrated that systemic inhibition of miR-15 by locked nucleic acid–modified anti-miRs reduces infarct size and cardiac remodeling and enhances cardiac function in response to myocardial infarction in mice. Cardiac expression and function are also described for another miR-16 family member, miR-195, which is upregulated during cardiac hypertrophy. Moreover, mice overexpressing miR-195 under the β-myosin heavy chain promoter develop fatal dilated cardiomyopathy (van Rooij et al. 2006). Recently, miR-195 was shown to be the most expressed miR in the heart between postnatal days 1 and 10. In particular, miR-195 is associated with cardiomyocyte postmitotic arrest due to the inhibition of several genes involved in cell cycle progression (Porrello et al. 2011). Furthermore, miR-195 and miR-497 are upregulated during aortic development, with subsequent downregulation of their target genes, including elastin (ELN), collagen 1a1 (Col1a1), and collagen 1a2 (Col1a2) (Ott et al. 2011) (Table 2). Finally, miR-103 and miR-107 are negative regulators of insulin sensitivity and may contribute to the etiology of diabetes. Moreover, global miR-103/107 silencing causes increased insulin signaling in both liver and adipose tissue (Trajkovski et al. 2011).

## Direction of Future Research

Dysfunction of the blood vascular endothelium is a major factor in the pathogenesis of micro- and macroangiopathy. Restoring EC function is fundamental in reestablishing vessel integrity and maintaining tissue perfusion. In the development of novel therapies aimed at preserving the EC layer and promoting reparative angiogenesis, the first challenge is to find a combination of factors able to prevent EC apoptosis and improve EC functional capacities. To achieve this, the regulation of gene networks should play a fundamental role. In this context, regulation of specific miRs or an entire family of miRs and consequently modulation of their target genes could lead to novel therapeutic approaches. In our study on miR-503, we showed the first example of a miR-based intervention normalizing postischemic reparative angiogenesis in diabetes. We demonstrated that antagonizing miR-503 using adenovirus-mediated local delivery of a competitive inhibitor (an miR-503 decoy) improved postischemic reparative neovascularization and blood flow recovery and restored expression of miR-503 target genes (Caporali et al. 2011). Because miR-15a, -15b, -16, -195, -424, -497, and -503 regulate overlapping lists of miR targets due to similarity in seed sequence, inhibition of the entire family of miRs could lead to a further benefit postischemic revascularization, including in diabetic patients.

However, the most advanced approach currently used to regulate miR levels in vivo is the use of anti-miRs. Anti-miRs are modified antisense oligodeoxynucleotides harboring the full or partial complementary reverse sequence of a mature miR. These oligodeoxynucleotides are able to reduce the endogenous levels of the miR, thus increasing expression of its mRNA targets. Recently, anti-miR technology has been used for miR-15 inhibition in the setting of ischemic heart disease (Porrello et al. 2011), and preclinical studies are ongoing at miRagen Therapeutics (http://www.miragentherapeutics.com) with the aim to commercialize an miR-15 inhibitor for the treatment of cardiovascular disorders.

Although there has been a significant increase in the number of patent application filings during the past 10 years and great excitement surrounding miRs as novel drugs, only one anti-miR compound has been included in clinical trials: Anti-miR-122 has been used to treat liver disease caused by hepatitis C virus (http://www.santaris.com). Further studies are necessary to clarify any “off-target” effect of miRs inhibitors, which may need to be addressed in order to fill the bench-to-bedside gap.

Finally, the discovery of miRs in biological fluids and the hypothetical possibility use of miRs as clinical diagnostic and prognostic biomarkers is an hot topic in the miR field. Further investigations of miR-503 as a possible biomarker for peripheral artery disease (PAD) in diabetic subjects are required. We plan to investigate the predictive potential of circulating miR-503 for leg ischemia in PAD patients. Larger numbers of patients and correlations with clinical outcomes need to be analyzed. Furthermore, measuring the level of other miR-16 family members in PAD patients without diabetes could help in the prediction of limb ischemia.

## Figures and Tables

**Table 1:** Sequences of the mature forms of miRs belonging to the extended miR-16 family^a^

miRs	First nucleotide of the mature miR sequence	Seed common region	Last nucleotide of the seed sequence	End of the mature miR sequence
hsa-miR-15a	U	AGCAGC	A	CAUAAUGGUUUGUG
hsa-miR-15b	U	AGCAGC	A	CAUCAUGGUUUACA
hsa-miR-16	U	AGCAGC	A	CGUAAAUAUUGGCG
hsa-miR-195	U	AGCAGC	A	CAGAAAUAUUGGCC
hsa-miR-503	U	AGCAGC	G	GGAACAGUUCUGCAG
hsa-miR-424	C	AGCAGC	A	AUUCAUGUUUUGAA
hsa-miR-497	C	AGCAGC	A	C ACUGUGGUUUGU

a Note that the last nucleotide of the seed region is a G only for miR-503.

**Table 2:** Extended miR-16 family in vascular biology and diabetes

miRNAs	Identified target genes	Stimuli inducing miR expression upregulation	References
miR-15b	Pdk4, Sgk1	Myocardial infarction	Hullinger et al. (2012)
miR-16	VEGFR2, FGFR1, VEGF	VEGF-A, bFGF	Chamorro-Jorganes et al. (2011)
miR-103/107	Caveolin-1	Type 2 diabetes	Trajkovski et al. (2011)
miR-195/497	ELN, Col1a1, Col1a2	Aortic development	Ott et al. (2011)
miR-195	Unknown	Cardiac hypertrophy	Van Rooij et al. (2006)
	Check1	Heart development	Porrello et al. (2011)
miR-424	CUL2	Ischemia	Ghosh et al. (2010)
	VEGFR2, FGF-R1, VEGF	VEGF-A, bFGF	Chamorro-Jorganes et al. (2011)
	MEK, CCNE1	Hemangioma	Nakashima et al. (2010)
miR-503	CCNE1, cdc25A	Diabetes + critical limb ischemia	Caporali et al. (2011)
